# Congenital solitary osseous choristoma of the left lateral canthus: a case report

**DOI:** 10.1186/s12886-024-03403-y

**Published:** 2024-03-28

**Authors:** Hatim Najmi, Shaikha Aleid, Fatimah Badghaish, Yara Alnashwan

**Affiliations:** 1 Oculoplastic Division, Department of Ophthalmology, Dhahran Eye Specialist Hospital, Dhahran, Saudi Arabia; 2 Oculoplastic Division, Department of Ophthalmology, King Fahad University Hospital, Khobar, Saudi Arabia; 3 College of Medicine, Imam Abdulrahman Bin Faisal University, Dammam, Saudi Arabia; 4 Department of Pathology, College of Medicine, Imam Abdulrahman Bin Faisal University, Dammam, Saudi Arabia

**Keywords:** Eyelid tumor, Lateral canthus, Osseous choristoma, Osteoma cutis

## Abstract

**Background:**

An ocular osseous choristoma is a growth of mature, compact bone in the ocular or periocular soft tissue, and it is the rarest form of ocular choristoma, accounting for only 1.7% of all epibulbar choristomas.

**Case presentation:**

Herein we present the case of a 20-month-old girl who was referred to the oculoplasty clinic with a progressively growing mass in the left lateral canthus. It had been present since birth without ocular involvement. Upon examination the mass was firm with a smooth surface, measured 9 × 6 × 3 mm, and exhibited no episcleral attachment or ocular involvement. An excisional biopsy was performed, and the histopathological findings were consistent with osseous choristoma of the left lateral canthus.

**Conclusions:**

This report highlights the importance of considering osseous choristoma in the differential diagnosis of eyelid lesions, particularly those that have been present since birth. It also emphasizes the need for further studies investigating associations between osseous choristomas and ocular canthi.

## Background

Choristoma refers to histologically normal tissue that proliferates in an abnormal anatomical location. The term “osseous choristoma” refers to abnormal ossification that results in a solid solitary nodular lesion consisting of mature bone surrounded by periosteal fibrous tissue. Osseous choristomas are believed to be isolated congenital lesions, rather than being associated with other congenital anomalies [1].

Ocular choristomas are classified as limbal dermoids, dermolipomas, complex choristomas, and single-tissue choristomas, including osseous choristomas [2]. The osseous type of ocular choristoma involves the growth of mature, compact bone in the ocular or periocular soft tissue, and is the rarest form of ocular choristoma, accounting for only 1.7% of all epibulbar choristomas [2, 3]. The origin of osseous choristomas varies and includes different ocular or periocular tissues, with supratemporal episcleral locations being the most frequently reported sites [1, 2].

Herein we present the case of a 20-month-old girl with congenital solitary osseous choristoma originating from the left lateral canthus.

## Case presentation

A healthy 20-month-old Saudi girl was referred from a general pediatric clinic to an oculoplasty clinic with a slowly progressing mass in the lateral corner of the left eye. The mass had been present since birth and had gradually increased in size with developmental growth. The mother denied any history of trauma, pain, constitutional symptoms, other lesions, or changes in the lesion itself. According to the mother the lesion did not affect the patient’s vision, lacrimation, or quality of life.

The child had been delivered prematurely in week 36 by emergency cesarean section. Her birth weight was 1,900 g, and she had no history of birth injuries or congenital anomalies. She was healthy, fully immunized, meeting her age milestones, and growing normally. Prenatal and postnatal histories were unremarkable.

**Fig. 1:**
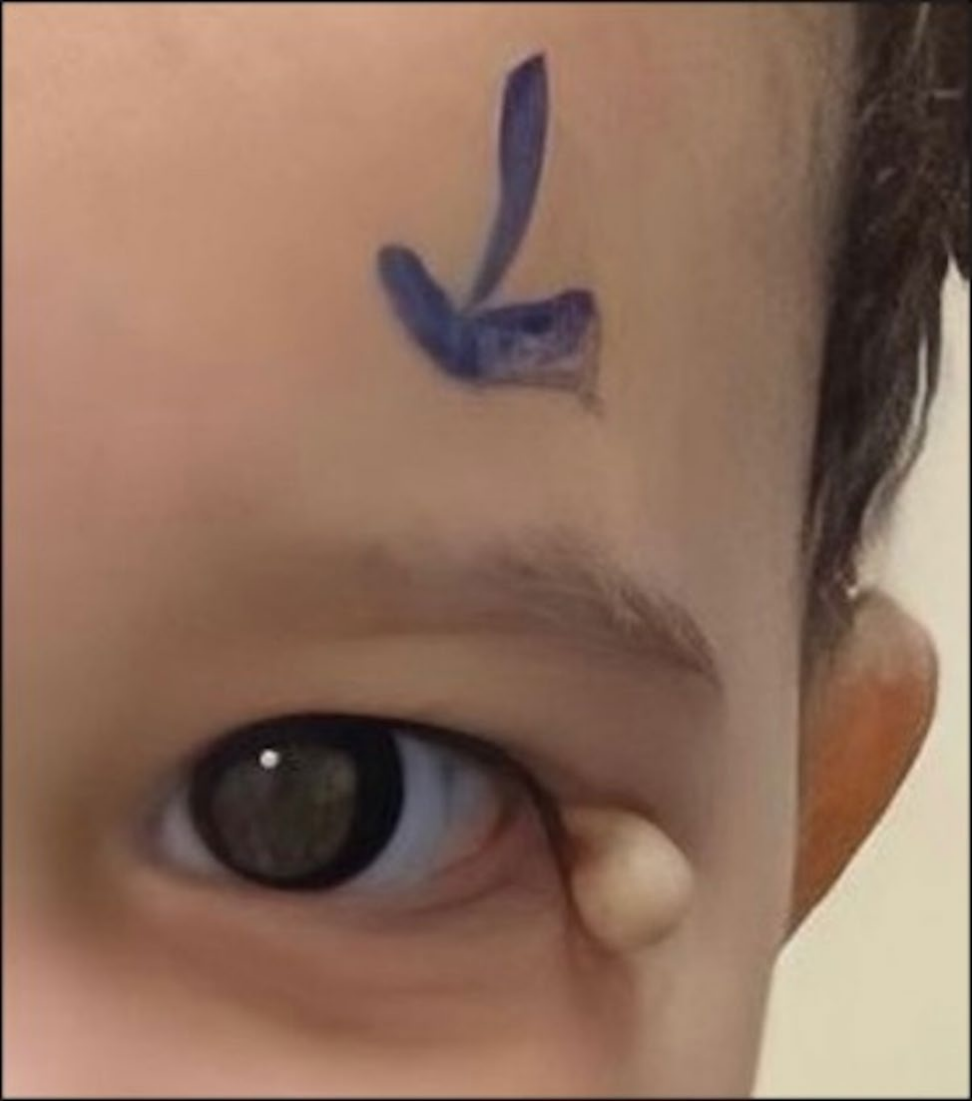
Lesion occupying the left lateral canthus

The patient’s vital signs were stable, and she appeared healthy with no dysmorphic facial features. Visual acuity examination revealed fix and follow in both eyes. External ophthalmic examination revealed a solitary well-defined pedunculated yellowish-white mobile mass measuring 9 × 6 × 3 mm occupying the left lateral canthus of the left eye, with a connecting stalk, firm consistency, and smooth surface (Fig. 1). The mass was not attached to the orbital rim, and no ulceration, telangiectasia, eyelash loss, signs of inflammation, or other skin lesions were evident. Orbital imaging was not performed because of the external location of the mass and non-adherence to the underlying tissue. Due to the cosmetic appearance and growth noted by the family, they elected to have the lesion excised. An excisional biopsy was planned, and written informed consent was obtained from the patient’s family.

The procedure was performed under general anesthesia. The patient was prepared and draped under sterile conditions. The base of the lesion was then marked, and a local anesthetic solution of 1% lidocaine with 1:200,000 epinephrine was infiltrated to ensure good hemostasis. The skin over the marked area was incised using a #15 blade, and careful dissection was performed until a single piece of skin with firm underlying tan tissue was removed. The skin over the area was closed using 6 − 0 rapid vicryl sutures and skin adhesive tape (Steri-Strips™). The patient was in good physical condition when she left the operating room and was discharged from the hospital with instructions to have tobramycin-dexamethasone ointment applied twice daily for 2 weeks.

**Fig. 2:**
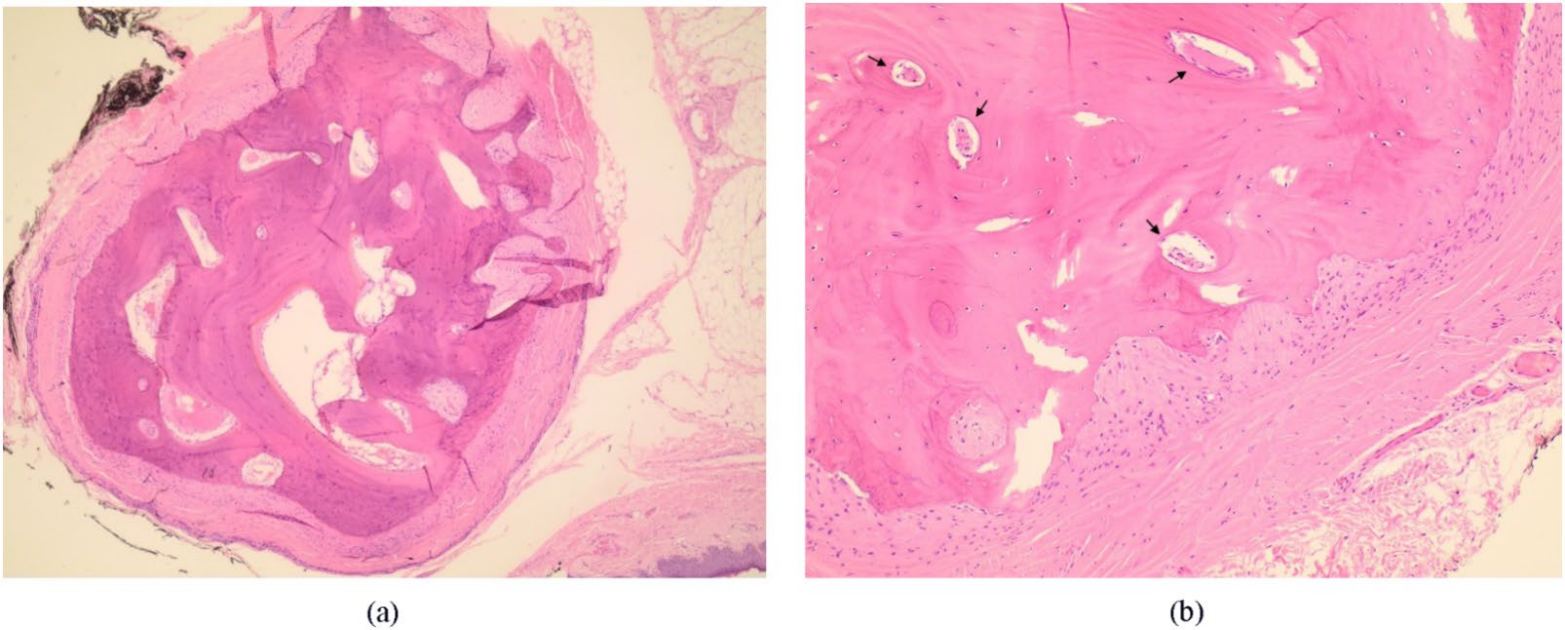
Hematoxylin and eosin-stained sections of the osseous choristoma. (**a**) Low magnification microscopy image depicting well-circumscribed mature bone surrounded by fibrous stroma, situated beneath the subcutaneous tissue (original magnification, ×40). (**b**) Higher magnification microscopy image depicting mature bone containing concentric osteocytes, around a central canal of fibrovascular tissue (black arrows, original magnification, ×100)

**Fig. 3:**
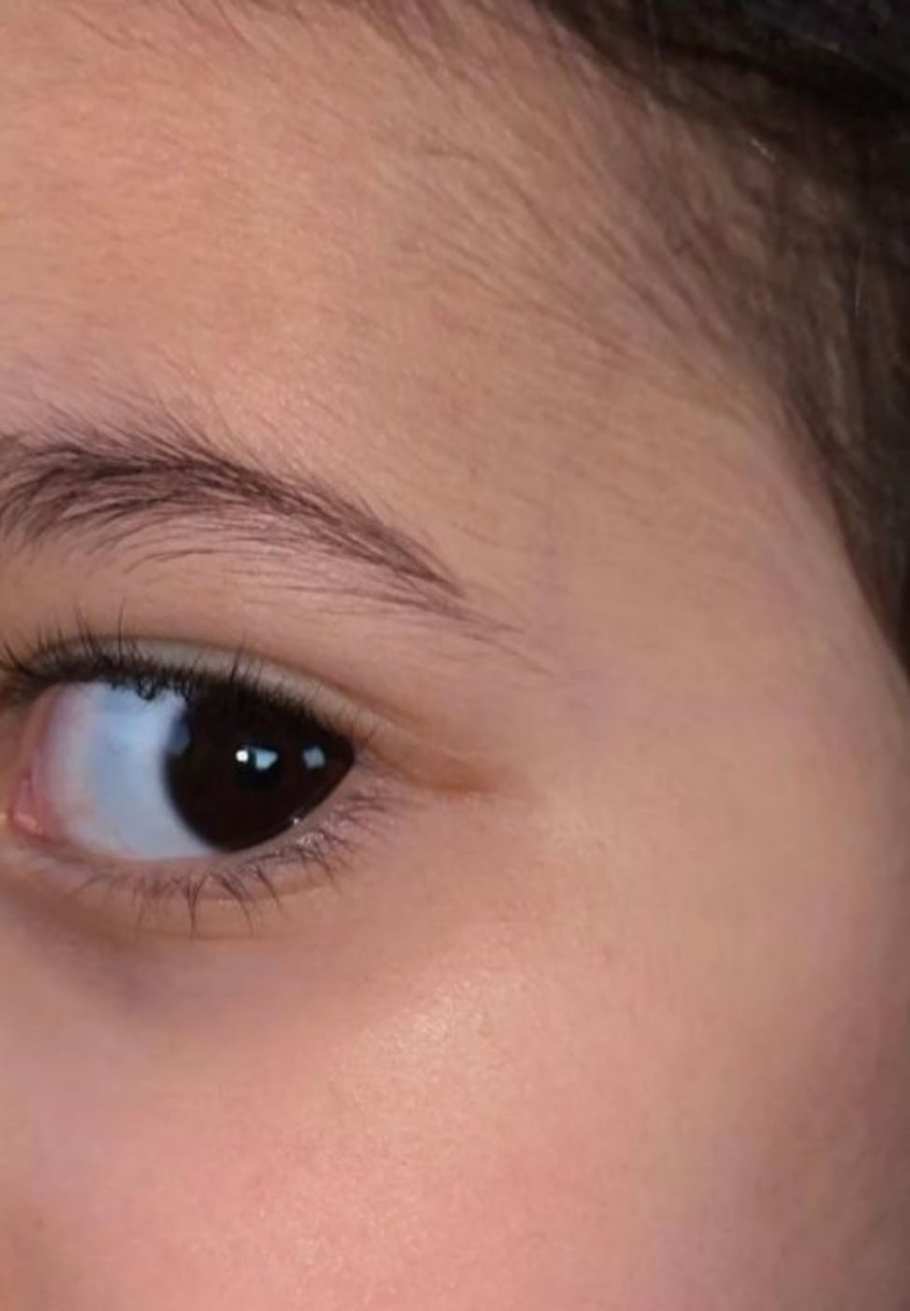
Appearance of the surgical site 6 months postoperatively

Histopathologic examination revealed a well-circumscribed, mature bony lesion beneath the subcutaneous tissue, with osteocytes in lacunae arranged concentrically around a central canal of fibrovascular tissue (Fig. 2). The overlying epidermis, dermis, and subcutaneous tissue appeared unremarkable. These findings are consistent with osseous choristoma. Six months postoperatively the patient was doing well, and there was no recurrence of the lesion in the same area or any other areas. The appearance of the surgical site was acceptable (Fig. 3).

## Discussion

This report describes a congenital solitary osseous choristoma of the left lateral canthus, a rare condition that is seldom reported [4–8]. The term “osteoma cutis” is used when the ossification involves cutaneous layers. However, “osteoma cutis” and “osseous choristoma” have frequently been used interchangeably in the literature [4–9]. While the exact pathophysiology of osseous choristoma remains unclear, one theory suggests that it involves the migration of mesenchymal cells to abnormal sites, and their subsequent differentiation into osteoplastic cells [9].

Osseous choristoma may originate from different ocular or periocular tissues, including choroid, episclera, conjunctiva, and extraocular muscles [2]. The formation of osseous choristoma from the lateral canthus is considered extremely rare, however, and only a few cases have been reported [4–8]. In most published cases, the masses were present since birth, without ocular involvement, and were located in the lateral canthus of the eyelid skin. In one case, a medial canthal location was reported [10]. Gupta et al. [5] published two cases of left lateral canthal masses that were similar to the current case in terms of size, shape, and pedunculated appearance, though in those cases there was adherence of the mass to the conjunctiva.

The diagnosis of osseous choristomas can be challenging because their clinical presentation varies depending on the location and size of the lesion, and they are often mistaken for other benign or malignant skin tumors [9, 11]. Histopathologic examination is required to confirm a diagnosis of osseous choristoma, and they exhibit mature bone formation surrounded by dense fibrous stroma with concentric osteocytes arranged around central canals containing fibrovascular tissue. Osteoblastic or osteoclastic activity is rarely present [3, 11].

The treatment of osseous choristoma depends on the location and size of the lesion, as well as the patient’s symptoms and cosmetic concerns. Small asymptomatic lesions that do not cause cosmetic concerns may not require treatment, but larger lesions causing functional or cosmetic problems may require surgical excision [9, 12]. In cases where the lesion is located in a cosmetically sensitive area, such as the face or neck, careful surgical planning is required to minimize scarring and achieve satisfactory cosmetic results [13]. The prognosis of osseous choristoma is generally excellent, with low recurrence rates reported in the literature, suggesting that the benign nature of the tumor can help reduce patient anxiety and avoid unnecessary interventions [9, 11].

## Conclusions

This report highlights the importance of considering osseous choristoma in the differential diagnosis of eyelid tumors, particularly in cases where the lesion has been present since birth. Further studies are required, to investigate associations between osseous choristoma and the lateral canthal area, which may explain the embryological basis and pathogenesis of this tumor.

## Data Availability

The datasets used during the current study are available from the corresponding author on reasonable request.
